# The impact of variants and vaccination on the mortality and resource utilization of hospitalized patients with COVID-19

**DOI:** 10.1186/s12879-022-07657-z

**Published:** 2022-08-22

**Authors:** Maria Stepanova, Brian Lam, Elena Younossi, Sean Felix, Mariam Ziayee, Jillian Price, Huong Pham, Leyla de Avila, Kathy Terra, Patrick Austin, Thomas Jeffers, Carey Escheik, Pegah Golabi, Rebecca Cable, Manirath Srishord, Chapy Venkatesan, Linda Henry, Lynn Gerber, Zobair M. Younossi

**Affiliations:** 1 Betty and Guy Beatty Center for Integrated Research, Inova Health System, Falls Church, VA USA; 2 Department of Medicine, Center for Liver Disease, Inova Fairfax Medical Campus, Falls Church, VA USA; 3 Medicine Service Line, Inova Health System, Falls Church, VA USA; 4 Betty and Guy Beatty Center for Integrated Research, Claude Moore Health Education and Research bldg., 3300 Gallows rd, Falls Church, VA 22042 USA

**Keywords:** Emerging diseases, Healthcare utilization, Multimorbidity, Pneumonia, Immunization, Public health

## Abstract

**Background:**

COVID-19 outcomes among hospitalized patients may have changed due to new variants, therapies and vaccine availability. We assessed outcomes of adults hospitalized with COVID-19 from March 2020–February 2022.

**Methods:**

Data were retrieved from electronic health medical records of adult COVID-19 patients hospitalized in a large community health system. Duration was split into March 2020–June 2021 (pre-Delta period), July–November 2021 (Delta period), and December 2021–February 2022 (Omicron period).

**Results:**

Of included patients (n = 9582), 75% were admitted during pre-Delta, 9% during Delta, 16% during Omicron period. The COVID-positive inpatients were oldest during Omicron period but had lowest rates of COVID pneumonia and resource utilization (p < 0.0001); 46% were vaccinated during Delta and 61% during Omicron period (p < 0.0001). After adjustment for demographics and comorbidities, vaccination was associated with lower inpatient mortality (OR = 0.47 (0.34–0.65), p < 0.0001). The Omicron period was independently associated with lower risk of inpatient mortality (OR = 0.61 (0.45–0.82), p = 0.0010). Vaccination and Omicron period admission were also independently associated with lower healthcare resource utilization (p < 0.05). Magnitudes of associations varied between age groups with strongest protective effects seen in younger patients.

**Conclusion:**

Outcomes of COVID-19 inpatients were evolving throughout the pandemic and were affected by changing demographics, virus variants, and vaccination.

**Key point:**

In this observational study of almost 10,000 patients hospitalized from March 2020–February 2022 with COVID-19, age and having multiple comorbidities remained consistent risk factors for mortality regardless of the variant. Vaccination was high in our hospitalized patients. Vaccination conveyed less severe illness and was associated with lower inpatient mortality.

**Supplementary Information:**

The online version contains supplementary material available at 10.1186/s12879-022-07657-z.

## Introduction

Since the first cases of SARS-CoV-2 infection have been reported in the United States in early 2020, there have been substantial changes in its rate of infectivity, severity, proclivity for specific organ system involvement, and responsiveness to different types of therapeutic interventions [1–3]. The burden of the disease has also changed substantially after multiple vaccines as well as preventive and anti-viral treatments became available.

In the mid-Atlantic region of the United States, three distinct peaks of SARS-CoV-2 infections and hospitalizations were observed: the first peak (the Wuhan wild type; March–May 2020), the second peak (B.1.617, or Delta variant, late summer 2021–late fall 2021) and the third peak (the BA.1, or Omicron variant, December 2021–February 2022). In fact, this pattern of infection spread was followed across most of the U.S. in 2020–2021 although exact timing varied between regions. The earliest studies indicated that patient clinical and demographic profile were major drivers of adverse outcomes so that older patients and those with comorbidities were at a significantly higher risk for hospitalization and mortality [4, 5].

Over the course of the pandemic, new treatments, including anti-viral agents, monoclonal antibodies and vaccines have been developed to reduce rates of infection, hospitalization, and mortality [4–10]. Awareness of patients’ demographic and clinical comorbidities throughout the pandemic have also been useful in clinical management and limiting the impact on healthcare systems [11]. Nonetheless, as of early 2022, the COVD-19 pandemic trajectory still lacks a clear path forward and healthcare systems constantly have to reevaluate their current and future clinical management strategies; in this context, it is important to assess the rates and risk factors for hospitalization throughout different phases of the pandemic to ensure public health policy and healthcare resources are optimized for different scenarios.

Our aim was to build upon our previous work [4] conducted during the first two phases of the pandemic by evaluating the outcomes of hospitalized patients with COVID-19 as the pandemic has now moved through the multiple stages, this time with a targeted focus on outcomes related to the dominant variants and patients’ vaccination status.

## Methods

As noted in our prior study, data were collected from electronic medical records (EMRs) of adults (≥ 18 years) with a diagnosis of COVID-19 (ICD-10 code U07.1) who were hospitalized in our health system [4]. The data for this study were collected over a longer period of time and are inclusive of March 5th, 2020 to February 5th, 2022. All patients had to have a discharge status at the time of the data acquisition. The health system includes five hospitals with a total of 1800 licensed acute care beds.

### Study definitions

Charlson comorbidity index (CCI) and Elixhauser comorbidity index (ECI) scores which are used in clinical practice to predict comorbid patients’ risk of mortality were calculated from the EMRs [4]. Obesity was defined as a body mass index (BMI) ≥ 30, morbid obesity was defined as a BMI ≥ 40. Low oxygen saturation was defined as ≤ 90% at admission with or without the use of supplemental oxygen. COVID-19 pneumonia was defined as the presence of the respective ICD-10 code (J12.82) for the admission.

History of vaccination for COVID-19 was collected from EMRs and verified via chart review. Patients were considered vaccinated if they have received at least one dose > 10 days prior to the admission. Details of vaccination, including the kind of vaccine and a number of doses, were available for roughly half of vaccinated patients. Using those, patients were considered under-vaccinated if they received only one dose of a two-dose regimen, fully vaccinated if they received both doses of a two-dose regimen or a single-dose regimen, and boosted if they received at least one additional dose after being fully vaccinated.

The study duration was split based on the most prevalent SARS-CoV-2 variant at the time as follows: March 2020–June 2021 (pre-Delta), July–November 2021 (predominantly-Delta), and December 2021–February 2022 (predominantly-Omicron) (Additional file 1: Fig. S1).

Study outcomes included COVID pneumonia, inpatient mortality, and inpatient resource utilization such as length of hospital stay (LOS in days), admission to the intensive care unit (ICU), and the use of mechanical ventilation. In our EMRs, patients who die while hospitalized are considered to be discharged from the hospital as of their date of death.

### Statistical analysis

Patients’ parameters were summarized as N (%) or mean (± SD). Parameters were compared between pre-defined comparison groups using chi-square or Kruskal–Wallis tests for categorical or continuous parameters, respectively. For patients with multiple inpatient admissions with COVID positivity, demographic and clinical parameters collected at the time of the first admission were used, along with the outcome of the most recent admission, and the total cumulative resource utilization across all admissions. Logistic regression models were used to identify parameters associated with the outcomes. Two-sided p-values < 0.05 were considered statistically significant.

SAS 9.4 (SAS Institute, Cary, NC, USA) was used for all analyses. The study was granted a waiver of consent and an exemption status by the Inova Health System’s Institutional Review Board given that all data were deidentified and analyzed anonymously. All methods were performed in accordance with the Declaration of Helsinki.

## Results

We included 9528 COVID-positive hospitalized patients. Of those, 7112 (75%) were admitted during the pre-Delta period, 860 (9%) during the Delta period, and 1556 (16%) during the Omicron period (Additional file 1: Fig. S1). The clinical profile and outcomes of our inpatients with COVID positivity during the pre-Delta period have been previously published [4].

### Comparison of different periods of the COVID-19 pandemic

The clinical and demographic variables as well as outcomes of COVID-19 patients hospitalized during the three variant peaks are summarized in Table 1. The COVID-positive inpatients were the oldest during the Omicron period with the highest comorbidity rates (p < 0.0001) (Table 1). Despite this, COVID disease severity as measured by the oxygen saturation at time of admission and the presence of COVID pneumonia was the lowest during the Omicron period (p < 0.0001) (Table 1). The proportion of vaccinated COVID-positive inpatients was < 1.5% during the pre-Delta period, 46% during Delta period, and 61% during Omicron period (p < 0.0001).

**Table 1:** Comparison of COVID-positive inpatients by the period of admission

	Pre-delta	Delta	Omicron	P*	All
N	7112	860	1556		9528
Age, years	57.7 ± 19.3	58.8 ± 19.3	61.0 ± 19.9	< 0.0001	58.3 ± 19.4
Male sex	3630 (51.0%)	458 (53.3%)	783 (50.3%)	0.37	4871 (51.1%)
BMI	30.0 ± 7.4	29.9 ± 7.8	29.0 ± 7.9	< 0.0001	29.8 ± 7.5
Obesity (BMI > 30)	2935 (43.4%)	359 (43.3%)	559 (38.0%)	0.0007	3853 (42.5%)
Morbid obesity (BMI > 40)	573 (8.5%)	74 (8.9%)	126 (8.6%)	0.90	773 (8.5%)
Comorbidities					
Charlson’s comorbidity index (CCI)	3.78 ± 3.57	4.25 ± 3.85	5.05 ± 3.90	< 0.0001	4.03 ± 3.68
Myocardial infarction	606 (8.5%)	115 (13.4%)	226 (14.5%)	< 0.0001	947 (9.9%)
Congestive heart failure	1058 (14.9%)	180 (20.9%)	390 (25.1%)	< 0.0001	1628 (17.1%)
Peripheral vascular disease	749 (10.5%)	112 (13.0%)	254 (16.3%)	< 0.0001	1115 (11.7%)
Cerebrovascular disease	894 (12.6%)	139 (16.2%)	279 (17.9%)	< 0.0001	1312 (13.8%)
Dementia	882 (12.4%)	106 (12.3%)	250 (16.1%)	0.0004	1238 (13.0%)
Chronic pulmonary disease	1514 (21.3%)	214 (24.9%)	458 (29.4%)	< 0.0001	2186 (22.9%)
Connective tissue/rheumatic disease	203 (2.9%)	39 (4.5%)	77 (4.9%)	< 0.0001	319 (3.3%)
Peptic ulcer disease	197 (2.8%)	27 (3.1%)	72 (4.6%)	0.0007	296 (3.1%)
Mild liver disease	675 (9.5%)	96 (11.2%)	173 (11.1%)	0.07	944 (9.9%)
Diabetes without complications	1542 (21.7%)	139 (16.2%)	217 (13.9%)	< 0.0001	1898 (19.9%)
Diabetes with complications	1104 (15.5%)	172 (20.0%)	361 (23.2%)	< 0.0001	1637 (17.2%)
Paraplegia and hemiplegia	228 (3.2%)	34 (4.0%)	68 (4.4%)	0.05	330 (3.5%)
Renal disease	1399 (19.7%)	211 (24.5%)	490 (31.5%)	< 0.0001	2100 (22.0%)
Non-metastatic cancer	355 (5.0%)	60 (7.0%)	130 (8.4%)	< 0.0001	545 (5.7%)
Moderate/severe liver disease	89 (1.3%)	3 (0.3%)	34 (2.2%)	0.0005	126 (1.3%)
Metastatic carcinoma	123 (1.7%)	17 (2.0%)	55 (3.5%)	< 0.0001	195 (2.0%)
AIDS	24 (0.3%)	1 (0.1%)	5 (0.3%)	0.55	30 (0.3%)
Elixhauser’s comorbidity index (ECI)	10.4 ± 11.5	12.7 ± 12.3	14.2 ± 12.7	< 0.0001	11.2 ± 11.9
COVID-related findings					
O_2_ saturation at admission ≤ 90%	1610 (22.8%)	182 (21.2%)	260 (16.8%)	< 0.0001	2052 (21.7%)
COVID-19 pneumonia	2743 (38.6%)	655 (76.2%)	918 (59.0%)	< 0.0001	4316 (45.3%)
Coagulopathy	832 (11.7%)	146 (17.0%)	217 (13.9%)	< 0.0001	1195 (12.5%)
Vaccinated (at least 1 dose)	63 (1.3%)	372 (45.5%)	881 (60.6%)	< 0.0001	1316 (18.7%)
Inpatient resource utilization					
LOS, days	9.18 ± 10.68	9.70 ± 11.70	7.14 ± 6.23	< 0.0001	8.90 ± 10.22
Admitted to ICU	2042 (28.7%)	184 (21.4%)	239 (15.4%)	< 0.0001	2465 (25.9%)
LOS in ICU, days	9.75 ± 11.70	9.35 ± 16.33	5.15 ± 6.29	< 0.0001	9.27 ± 11.78
Invasive mechanical ventilation	838 (11.8%)	115 (13.4%)	118 (7.6%)	< 0.0001	1071 (11.2%)
ECMO	59 (0.8%)	13 (1.5%)	4 (0.3%)	0.0034	76 (0.8%)
Disposition					
Short-term care facility	63 (0.9%)	17 (2.0%)	15 (1.0%)	0.0097	95 (1.0%)
Long-term care facility	729 (10.3%)	102 (11.9%)	166 (10.7%)	0.33	997 (10.5%)
Home (including home healthcare)	5373 (75.5%)	618 (71.9%)	1189 (76.4%)	0.0344	7180 (75.4%)
Hospice (including home hospice)	158 (2.2%)	42 (4.9%)	65 (4.2%)	< 0.0001	265 (2.8%)
Expired	789 (11.1%)	81 (9.4%)	121 (7.8%)	0.0003	991 (10.4%)

The parameters are given as mean ± SD or N (%); missing entries not included in calculation of percentages

* BMI* body mass index, *CCI* Charlson’s comorbidity index (CCI), *ECI* Elixhauser’s comorbidity index, *LOS* length of stay

*By chi-square (categorical parameters) or Kruskal–Wallis (continuous parameters) non-parametric test

Resource utilization for inpatients with COVID positivity was the lowest during the Omicron period (Table 1). In particular, the mean LOS was 9.2 days (5th to 95th percentile range 2–28 days) pre-Delta, 9.7 (2–31) days during Delta, and 7.1 (2–20) days during Omicron periods (p < 0.0001). The rate of ICU use decreased from 29 to 21% to 15%, respectively (p < 0.0001), and the rate of mechanical ventilation use changed from 12 to 13% to 8% (p < 0.0001). All three resource utilization outcomes were also significantly different between the Delta and Omicron periods (all p < 0.001).

Inpatient mortality was the lowest during the Omicron period: 7.8% vs. 9.4% during Delta and 11.1% before Delta (p = 0.0003 across the three periods); however, the difference between Delta and Omicron periods was not significant (p = 0.16) (Table 1). Since pre-Delta period included the earliest months of the pandemic when optimal treatment protocols were still under development, its crude mortality rate of 11.1% includes 15.0% during March–May 2020 and 9.4% during June 2020–June 2021; the latter is identical to that seen during the Delta period (p > 0.10). On the other hand, the Omicron period includes first weeks of December 2021 when the Delta variant was still prevalent in our region so we additionally compared inpatient mortality rates between the months of December 2021 (8.8%) and January 2022 (7.2%, p = 0.26).

### Comparison of vaccinated and unvaccinated COVID-19 patients

During the Delta period, the vaccination rate among inpatients with COVID+ was 45.5%. During that period, in comparison to unvaccinated, vaccinated inpatients were substantially older (mean age 67 vs. 52 years), had more comorbidities (mean CCI 6.2 vs. 2.6, mean ECI 17.7 vs. 8.2), but despite this had less COVID pneumonia (67% vs. 85%) or low oxygen saturation at admission (13% vs. 27%) (all p < 0.0001). The crude mortality rate and LOS were numerically lower in the vaccinated group but did not reach statistical significance (Table 2).

**Table 2:** Comparison of COVID-positive inpatients during predominantly-Delta period (June–November 2021) and during predominantly-Omicron period (December 2021–January 2022) by vaccination status

	Vaccinated (at least 1 dose)	Not vaccinated	P*
Predominantly-Delta period			
N	372	445	
Age, years	67.1 ± 17.8	51.9 ± 17.4	< 0.0001
Male sex	209 (56.2%)	228 (51.2%)	0.16
Morbid obesity (BMI > 40)	28 (7.9%)	43 (9.8%)	0.34
Charlson’s comorbidity index (CCI)	6.17 ± 3.92	2.63 ± 2.99	< 0.0001
Elixhauser’s comorbidity index (ECI)	17.7 ± 13.1	8.22 ± 9.98	< 0.0001
O_2_ saturation at admission ≤ 90%	48 (12.9%)	122 (27.4%)	< 0.0001
COVID-19 pneumonia	248 (66.7%)	379 (85.2%)	< 0.0001
Coagulopathy	79 (21.2%)	62 (13.9%)	0.0059
LOS, days	10.6 ± 13.4	9.35 ± 10.46	0.16
Admitted to ICU	85 (22.8%)	86 (19.3%)	0.22
LOS in ICU, days	7.95 ± 16.15	11.6 ± 17.4	0.0142
Mechanical ventilation	46 (12.4%)	60 (13.5%)	0.64
Disposition: short-term care facility	5 (1.3%)	10 (2.2%)	0.34
Disposition: long-term care facility	65 (17.5%)	30 (6.7%)	< 0.0001
Disposition: home	246 (66.1%)	354 (79.6%)	< 0.0001
Disposition: hospice	26 (7.0%)	10 (2.2%)	0.0010
Expired	30 (8.1%)	41 (9.2%)	0.56
Predominantly-Omicron period			
N	881	572	
Age, years	63.4 ± 19.3	56.8 ± 19.9	< 0.0001
Male sex	447 (50.7%)	280 (49.0%)	0.51
Morbid obesity (BMI > 40)	68 (8.2%)	53 (9.7%)	0.32
Charlson’s comorbidity index (CCI)	5.87 ± 4.01	3.84 ± 3.53	< 0.0001
Elixhauser’s comorbidity index (ECI)	16.1 ± 13.0	11.5 ± 12.1	< 0.0001
O_2_ saturation at admission ≤ 90%	115 (13.1%)	133 (23.3%)	< 0.0001
COVID-19 pneumonia	461 (52.3%)	402 (70.3%)	< 0.0001
Coagulopathy	130 (14.8%)	72 (12.6%)	0.24
LOS, days	6.84 ± 5.52	7.79 ± 7.29	0.12
Admitted to ICU	125 (14.2%)	84 (14.7%)	0.79
LOS in ICU, days	4.79 ± 5.79	5.91 ± 7.35	0.26
Mechanical ventilation	56 (6.4%)	41 (7.2%)	0.54
Disposition: short-term care facility	10 (1.1%)	2 (0.3%)	0.11
Disposition: long-term care facility	106 (12.0%)	43 (7.5%)	0.0056
Disposition: home	659 (74.8%)	468 (81.8%)	0.0017
Disposition: hospice	37 (4.2%)	23 (4.0%)	0.87
Expired	69 (7.8%)	36 (6.3%)	0.27

The parameters are given as mean ± SD or N (%); missing entries not included in calculation of percentages

*BMI* body mass index, *CCI* Charlson’s comorbidity index (CCI), *ECI* Elixhauser’s comorbidity index, *LOS* length of stay

*By chi-square (categorical parameters) or Kruskal–Wallis (continuous parameters) non-parametric test

During the Omicron period, the vaccination rate among inpatients with COVID+ was 60.7%. Similar to the Delta period, vaccinated hospitalized patients during Omicron were older, albeit by a smaller magnitude, than unvaccinated (mean age 63 vs. 57 years), had more comorbidities (mean CCI 5.9 vs. 3.8, mean ECI 16.1 vs. 11.5) but less hypoxia (13.1% vs. 23.3%) or COVID pneumonia (52% vs. 70%) (all p < 0.0001). The crude mortality rate was lower but did not reach significance (Table 2).

The details of COVID vaccination were available for 43% of patients who had been vaccinated. Since COVID vaccine boosters were generally not available until mid-fall 2021, we only used the Omicron period to assess the impact of vaccination status on outcomes (Table 3). Of the entire subgroup of vaccinated patients who were hospitalized with COVID-19 during the predominantly-Omicron period with vaccination details available (n = 385), 13% were under-vaccinated, 69% were fully vaccinated without a booster, and 18% were fully vaccinated and had received a booster. Hospitalized patients with a booster were the oldest (mean age 67 years vs. 62 years in fully vaccinated without a booster vs. 53 years in under-vaccinated vs. 57 years in unvaccinated) with the highest comorbidity indices (mean CCI 6.8 vs. 6.1 vs. 5.2 vs. 3.8, respectively; mean ECI 18.5 vs. 17.0 vs. 15.0 vs. 11.5, respectively) (Table 3). The rates of hypoxia and COVID pneumonia were consistently lower in all vaccinated patients in comparison to the unvaccinated (both p < 0.01) but not different within the vaccinated group (p > 0.05). Similarly, there was no difference in inpatient resource utilization or mortality across the groups (p > 0.05) (Table 3).

**Table 3:** Comparison of COVID-positive inpatients during predominantly-Omicron period (December 2021–January 2022) by vaccination status

	Not vaccinated	Under-vaccinated	Fully vaccinated w/o booster	Fully vaccinated with booster	P*
N	572	49	266	70	
Age, years	56.8 ± 19.9	53.3 ± 17.6	62.1 ± 19.4	66.8 ± 18.8	< 0.0001
Male sex	280 (49.0%)	24 (49.0%)	140 (52.6%)	42 (60.0%)	0.31
Morbid obesity (BMI > 40)	53 (9.7%)	4 (8.3%)	21 (8.1%)	4 (6.0%)	0.71
Charlson’s comorbidity index (CCI)	3.84 ± 3.53	5.16 ± 3.82	6.14 ± 4.41	6.77 ± 3.77	< 0.0001
Elixhauser’s comorbidity index (ECI)	11.5 ± 12.1	15.0 ± 13.2	17.0 ± 14.6	18.5 ± 13.0	< 0.0001
O2 saturation at admission ≤ 90%	133 (23.3%)	8 (16.3%)	34 (12.8%)	9 (13.0%)	0.0016
COVID-19 pneumonia	402 (70.3%)	22 (44.9%)	153 (57.5%)	35 (50.0%)	< 0.0001
Coagulopathy	72 (12.6%)	5 (10.2%)	34 (12.8%)	12 (17.1%)	0.68
LOS, days	7.79 ± 7.29	6.71 ± 5.12	6.71 ± 5.75	6.74 ± 5.32	0.22
Admitted to ICU	84 (14.7%)	6 (12.2%)	41 (15.4%)	12 (17.1%)	0.89
LOS in ICU, days	5.91 ± 7.35	5.29 ± 4.53	4.24 ± 4.83	5.13 ± 8.12	0.53
Mechanical ventilation	41 (7.2%)	3 (6.1%)	18 (6.8%)	7 (10.0%)	0.81
Disposition: short-term care facility	2 (0.3%)	0 (0.0%)	2 (0.8%)	2 (2.9%)	0.08
Disposition: long-term care facility	43 (7.5%)	4 (8.2%)	22 (8.3%)	11 (15.7%)	0.14
Disposition: home	468 (81.8%)	40 (81.6%)	208 (78.2%)	46 (65.7%)	0.0148
Disposition: hospice	23 (4.0%)	1 (2.0%)	10 (3.8%)	3 (4.3%)	0.91
Expired	36 (6.3%)	4 (8.2%)	24 (9.0%)	8 (11.4%)	0.30

Under-vaccinated: only one dose of a two-dose regimen; fully vaccinated: both doses of a two-dose regimen or a single dose of a one-dose regimen; fully vaccinated with a booster: at least one additional dose after being fully vaccinated

The parameters are given as mean ± SD or N (%); missing entries not included in calculation of percentages

*BMI* body mass index, *CCI* Charlson’s comorbidity index (CCI), *ECI* Elixhauser’s comorbidity index, *LOS* length of stay

*By chi-square (categorical parameters) or Kruskal–Wallis (continuous parameters) non-parametric test

### Independent predictors of inpatient mortality and resource utilization

In multivariate analysis which included the entire study duration, independent predictors of inpatient mortality among COVID-positive patients included older age, male sex, morbid obesity, and a higher multi-morbidity score (all p < 0.0001) (Table 4). After adjustment for those, we found that being vaccinated was associated with a reduction of inpatient mortality by approximately half [OR (95%) = 0.47 (0.34–0.65) (p < 0.0001)]. In the same model, there was no independent association of mortality risk with being admitted during the Delta period (p = 0.47 with reference to pre-Delta) while admissions during the predominantly-Omicron period were additionally associated with a lower risk of inpatient mortality (OR = 0.61 (0.45–0.82), p = 0.0010) (Table 4). Similar observations were made when the model was adjusted for individual comorbidities rather than the composite comorbidity index (Additional file 1: Table S1).

**Table 4:** Multivariate analysis of independent predictors of inpatient mortality and resource utilization among COVID-positive patients

Outcome	Predictor	Adjusted OR (95% CI)^a^	p
Inpatient mortality	Age, per year	1.039 (1.033–1.046)	< 0.0001
	Male sex (ref: female)	1.51 (1.26–1.81)	< 0.0001
	Morbid obesity	1.99 (1.44–2.76)	< 0.0001
	ECI, per 1 point	1.06 (1.05–1.07)	< 0.0001
	Vaccinated	0.47 (0.34–0.65)	< 0.0001
	Delta (ref: pre-delta)	0.89 (0.64–1.23)	0.47
	Omicron (ref: pre-omicron)	0.61 (0.45–0.82)	0.0010

Outcome	Predictor	Beta ± SE^a^	p
Length of inpatient stay, days	Age, per year	0.009 ± 0.007	0.21
	Male sex (ref: female)	1.85 ± 0.24	< 0.0001
	Morbid obesity	1.09 ± 0.44	0.0126
	ECI, per 1 point	0.245 ± 0.012	< 0.0001
	Vaccinated	− 1.97 ± 0.41	< 0.0001
	Delta (ref: pre-delta)	0.49 ± 0.42	0.24
	Omicron (ref: pre-omicron)	− 2.20 ± 0.39	< 0.0001

Outcome	Predictor	Adjusted OR (95% CI)^a^	p
Admission to ICU	Age, per year	0.999 (0.996–1.003)	0.75
	Male sex (ref: female)	1.71 (1.52–1.91)	< 0.0001
	Morbid obesity	1.62 (1.33–1.97)	< 0.0001
	ECI, per 1 point	1.045 (1.039–1.051)	< 0.0001
	Vaccinated	0.69 (0.55–0.86)	0.0009
	Delta (ref: pre-delta)	0.53 (0.43–0.65)	< 0.0001
	Omicron (ref: pre-omicron)	0.32 (0.26–0.39)	< 0.0001
Mechanical ventilation	Age, per year	0.991 (0.986–0.996)	0.0005
	Male sex (ref: female)	1.84 (1.56–2.17)	< 0.0001
	Morbid obesity	1.55 (1.18–2.04)	0.0018
	ECI, per 1 point	1.070 (1.063–1.078)	< 0.0001
	Vaccinated	0.57 (0.43–0.77)	0.0002
	Delta (ref: pre-delta)	1.15 (0.88–1.50)	0.31
	Omicron (ref: pre-omicron)	0.50 (0.38–0.66)	< 0.0001

*ECI* Elixhauser’s comorbidity index, *OR* odds ratio

^a^Logistic (for binary outcomes) or generalized linear (for continuous outcomes) regression model

Other study outcomes were also found to be associated with being vaccinated and/or period of admission. In particular, both being vaccinated and being admitted during the Omicron period were independently associated with a shorter length of inpatient stay: beta = − 2.0 and − 2.2 days, respectively (both p < 0.0001) (Table 4). Similar findings were reported for the rates of ICU admission and mechanical ventilation use for which both being vaccinated and being admitted during the Omicron period were found to be associated with superior outcomes (all OR < 1.0, all p < 0.0001) (Table 4). On the other hand, for patients with vaccination details available, we found no significant association of having received a booster with any inpatient outcome including the risk of COVID pneumonia or inpatient death (all p > 0.10 with reference to being fully vaccinated without a booster).

When the study periods were analyzed separately, we found that being vaccinated was independently associated with a substantially lower risk of having COVID pneumonia among inpatients with COVID positivity during both Delta and Omicron periods: OR = 0.29 and 0.36, respectively (both p < 0.0001 after adjustment for age, sex, and comorbidities) (Additional file 1: Table S2). On the other hand, the association of vaccination with inpatient mortality was only observed during the Delta period (OR = 0.36, p = 0.0010). In addition, there was an association of being vaccinated with a shorter LOS during all periods, and with a lower risk of mechanical ventilation during the Delta period (p < 0.05) (Additional file 1: Table S2).

### Predictors of COVID-19 outcomes by age group

Since both outcome of COVID-19 disease and efficacy of COVID vaccines may vary depending on individuals’ age, we additionally assessed the association of the period of admission with inpatient outcomes stratified by patients’ age. In the subgroup of COVID-positive patients admitted during the Delta and Omicron periods, 37% were < 55 years of age, 17% were 55–64 years old, 18% were 65–74 years old, 16% were 75–84 years old, and 11% were at least 85 years old (Additional file 1: Table S3).

In multivariate logistic regression models adjusted for age, sex, and comorbidities, we found that the risk of COVID pneumonia was most strongly associated with vaccination in the younger age groups: OR = 0.27, 0.33, and 0.36 for the < 55, 55–64, and 65–74 age groups, respectively (all p < 0.0001) (Table 5). The association with inpatient mortality from COVID-19 with vaccination was only significant in the youngest patient group (OR = 0.37, p = 0.0496) and not significant in all other age groups (p > 0.05) (Table 5). The association of being admitted during the Omicron period (reference: Delta period) with the outcomes was again the strongest for the youngest patients (both p < 0.05 for the < 55 age group) and not present for patients older than 75 (all p > 0.10) (Table 5).

**Table 5:** Independent association of vaccination and period of admission with outcomes in COVID-positive patients by age group (predominantly-Delta and -Omicron periods only)

Outcome	Age group	In vaccinated	In non- vaccinated	p	OR for vaccinated vs. non-vaccinated (95% CI)^a^	p	OR for omicron vs. delta (95% CI)^a^	p
COVID pneumonia in hospitalized COVID-positive patients	< 55	138 (40.6%)	368 (71.7%)	< 0.0001	0.27 (0.19–0.38)	< 0.0001	0.31 (0.22–0.44)	< 0.0001
	55–65	139 (64.1%)	152 (86.4%)	< 0.0001	0.33 (0.19–0.57)	< 0.0001	0.37 (0.20–0.68)	0.0013
	65–75	156 (59.8%)	126 (80.8%)	< 0.0001	0.36 (0.22–0.59)	< 0.0001	0.73 (0.46–1.16)	0.18
	75–85	153 (60.5%)	85 (77.3%)	0.0020	0.41 (0.24–0.71)	0.0014	1.15 (0.71–1.86)	0.57
	85+	123 (67.6%)	50 (80.6%)	0.0505	0.49 (0.23–1.04)	0.06	0.76 (0.39–1.48)	0.42
Inpatient mortality in COVID-positive patients	< 55	7 (2.1%)	18 (3.5%)	0.22	0.37 (0.14–1.00)	0.0496	0.36 (0.15–0.88)	0.0241
	55–65	15 (6.9%)	16 (9.1%)	0.43	0.46 (0.20–1.06)	0.07	0.68 (0.30–1.55)	0.36
	65–75	31 (11.9%)	19 (12.2%)	0.93	0.95 (0.50–1.81)	0.87	0.50 (0.27–0.93)	0.0296
	75–85	27 (10.7%)	17 (15.5%)	0.20	0.52 (0.25–1.06)	0.07	1.06 (0.51–2.22)	0.87
	85+	19 (10.4%)	7 (11.3%)	0.85	0.89 (0.32–2.50)	0.82	2.45 (0.82–7.34)	0.11

^a^Adjusted for age, sex, Elixhauser’s comorbidity index (ECI), and morbid obesity in a logistic regression model

## Discussion

We assessed the impact of the COVID-19 pandemic and its association with major variants of SARS-CoV-2 as well as vaccination status among those hospitalized during the distinct peaks of hospitalizations in our region. This study builds on our previous data where we noted that inpatient mortality was the highest in the earliest months of the pandemic, primarily driven by older age and presence of multi-morbidities but as newer treatment protocols, novel antiviral regimens, and effective vaccines emerged, inpatient mortality and resource utilization decreased [4, 11–19]. In fact, our previous focused on the clinical predictors of outcomes for patients hospitalized with SARS-CoV-2 infection without specific assessment of the impact of vaccination or variants. Since then, accumulating evidence suggested that both variants and vaccinations may have an impact on the outcomes. Therefore, the current analysis of outcomes of COVID-19 patients was extended to include not only clinical factors but also the period when a particular SARS-CoV-2 variant is dominant as well as history of vaccination.

Our current data analysis showed that, regardless of a specific variant, several risk factors for inpatient mortality with COVID-19 remained consistent. These risks included advanced age and multi-morbidity. After adjustment for these factors and exclusion of the first months of the pandemic potentially affected by suboptimal clinical protocols for managing COVID-19 disease, we did not find major differences in inpatient mortality between the pre-Delta and Delta periods. Notably, the so-called pre-Delta period included multiple variants of SARS-CoV-2 but it was the original Wuhan variant that was responsible for the highest peak of infections in our region [20]. A more detailed analysis of our patients’ outcomes during the pre-Delta period has been published [4].

In this study, we found that the clinical profile of hospitalized patients with COVID-19 changed over the different periods. Specifically, patients who were admitted during the pre-Delta period were, on average, the youngest and had the lowest comorbidity scores but these differences did not seem to translate into superior outcomes. In contrast, during the Omicron surge, COVID-19 patients were the oldest and had the highest comorbidity scores. Despite this, these patients were less likely to have hypoxia at admission or COVID-19 pneumonia among their inpatient diagnoses while experiencing the shortest LOS, lower use of the ICU, mechanical ventilation, and ECMO, and being more likely to be discharged home. They also had the lowest inpatient mortality rate. These differences in outcomes can be partially explained by the expansion of knowledge and improved treatments for COVID-19, development of ambulatory management, and possibly increasing vaccination rates [8–11, 18].

To assess the interaction of variants and vaccination, we analyzed our data by vaccination status during periods when an opportunity to be vaccinated was widely available; that coincided with the periods when the Delta and Omicron variants were most commonly observed in our community. Overall, we found that hospitalized patients who were vaccinated experienced less severe respiratory distress (lower rates of hypoxia and pneumonia). Due to age and multi-morbidity biases between vaccinated and unvaccinated patients, there was no discernible difference in crude mortality rates by univariate analyses. This suggests that, for inpatient mortality, being vaccinated counter-weighed an approximately 15-year age difference observed during Delta and an approximately 6-year age difference during Omicron variant. On the other hand, in multivariate analysis which included adjustment for these confounders, being vaccinated was independently associated with a lower risk of having COVID pneumonia and a shorter LOS during both periods, lower risk of mechanical ventilation and lower inpatient mortality during the Delta period.

Consistent with other reports, the benefit of vaccination for inpatients with COVID positivity was also evident during the Omicron period albeit not as strong as observed during the Delta period [9, 10]. We found that over 60% of our inpatient population during the Omicron surge were vaccinated and, since these were the oldest group of patients to date, we suspect that they may have experienced a waning of vaccine-induced immunity as they were more likely to have received their vaccination earlier during the pandemic. We also found a limited booster uptake although those who were boosted were the very oldest and sickest of patients. Even though we did not detect any association of the vaccine booster with superior inpatient outcomes, the effect of the booster might have manifested via prevented hospitalizations as the percent of vaccinated and boosted patients hospitalized during the Omicron period (18%) was substantially lower than that of approximately 40–50% of the general population in our region [20].

Our multivariate analysis was in concordance with prior reports that Omicron variant itself may be less lethal than either Delta variant or its predecessors [21]. Indeed, we found that Omicron variant led to a 40% reduction in inpatient mortality, a 70% reduction in the risk of admission to the ICU, a 50% reduction in being mechanically ventilated, and a reduction in the hospital LOS of approximately 2 days.

Importantly, all the above associations were observed for the entire cohort of hospitalized COVID positive patients even after adjustment for age. However, it is plausible that the magnitude of the effect of vaccination and/or SARS-CoV-2 variants may be different for patients of different ages. For this purpose, we stratified our patients by age and evaluated the association of vaccination and the SARS-CoV-2 variants with the risk of having COVID-19 pneumonia and mortality. We found that vaccination was protective against COVID-19 pneumonia for all ages but the magnitude of the protection decreased with age (73% reduction in risk in those < 55 years to 51% reduction in risk for those > 84 years old). In fact, the effect of vaccination on inpatient mortality was less pronounced in older patients where the main predictor of the outcome remained the presence of multimorbidity. The relative protective effect of Omicron variant was noted in younger patients but not observed in those of 75 years of age or older. These results suggest that adverse inpatient outcomes from COVID-19 are primarily carried by elderly people with a high number of comorbid medical conditions even in the era of vaccination and antiviral regimens. Future treatment strategies for COVID-19 need to focus on managing the disease in the presence of multimorbidity and prevention of hospitalization via early treatment.

## Limitations

The study was based on the use of EMR so the data were susceptible to all biases that come from a retrospective observational study including our inability to draw causal relationships from the findings. Also, in this study, we projected the most dominant SARS-CoV-2 variant onto the entire patient sample admitted during the respective period of time rather than confirm it via genotyping at the patient level. Vaccination details were available only for a subset of vaccinated patients so some hypotheses remained underpowered. The presence of COVID-19 pneumonia was ascertained using the ICD code and this approach may miss cases of severe COVID-19 manifested via other complications; also, the respective ICD code was introduced relatively late during the pandemic so the condition could not be consistently evaluated during most of the pre-Delta period. The vaccination rate in our region is substantially higher than the U.S. average so our results regarding resource utilization may be not fully generalizable to other regions with lower vaccine uptake. On the other hand, our area of service is composed of primarily suburban counties, the kind the majority of the U.S. population currently lives in [22], which should improve generalizability of our findings. The data for patients who were not hospitalized were not available so the effect of the vaccines and variants on the risk of hospitalization could not be accurately evaluated. Post-discharge mortality was also not available.

## Conclusion

In this study of a cohort of almost 10,000 patients hospitalized with COVID-19, we found that the outcomes of COVID-19 inpatients were evolving over time. While predictors of inpatient mortality remained consistent with prior reports and included older age, being male, having morbid obesity, and multiple comorbidities, being vaccinated generally improved inpatient outcomes. The protective effect of vaccination was observed across all patient subgroups but was less pronounced in patients of advanced age. Omicron variant also seems to be a less lethal variant although with a weaker effect in the older patients. Despite this, the majority of inpatients with COVID-19 were vaccinated so near-complete elimination of healthcare resource utilization associated with SARS-CoV-2 infection is unlikely even in the presence of near-universal vaccine uptake. In order to further limit the burden of the disease, research needs to continue on whether management of medical comorbidities and/or early treatment of those infected with highly effective antivirals can further decrease COVID-19-associated severe disease, hospitalizations, and deaths.

## Supplementary Information

**Additional file 1: Table S1.** Independent predictors of inpatient mortality among COVID-positive patients adjusted for individual comorbidities. **Table S2. **Independent predictors of inpatient outcomes for COVID-positive patients by the period of admission. **Table S3.** COVID-19 inpatients by the age group and the period of admission. **Figure S1.** Distribution of COVID-positive admissions by calendar month. 

## Data Availability

The datasets used and/or analyzed during the current study are available from the corresponding author on reasonable request.

## Abbreviations

SARS-CoV-2: Severe acute respiratory syndrome coronavirus 2
EMR: Electronic medical record
BMI: Body mass index
CCI: Charlson’s comorbidity index
ECI: Elixhauser’s comorbidity index
qSOFA: Quick Sequential Organ Failure Assessment
ICU: Intensive care unit
ECMO: Extracorporeal membrane oxygenation
OR: Odds ratio
