# Supplementary material for: The impact of variants and vaccination on the mortality and resource utilization of hospitalized patients with COVID-19
Source: BMC Infect Dis. 2022 Aug 22;22:702. doi: 10.1186/s12879-022-07657-z (PMC9394045; doi:10.1186/s12879-022-07657-z)

**Table S1**. Independent predictors of inpatient mortality among COVID-positive patients adjusted for individual comorbidities.

| Predictor | OR (95% CI) | p |
| --- | --- | --- |
| Age, per year | 1.047 (1.04 – 1.054) | <.0001 |
| Male sex (ref: female) | 1.62 (1.35 – 1.93) | <.0001 |
| Morbid obesity | 1.90 (1.38 – 2.61) | <.0001 |
| Myocardial Infarction | 1.27 (0.99 – 1.63) | 0.06 |
| Congestive heart failure | 1.23 (0.99 – 1.54) | 0.07 |
| Peripheral vascular disease | 0.86 (0.67 – 1.10) | 0.22 |
| Cerebrovascular disease | 1.20 (0.96 – 1.51) | 0.11 |
| Dementia | 1.37 (1.10 – 1.71) | 0.0053 |
| Chronic pulmonary disease | 0.97 (0.79 – 1.18) | 0.73 |
| Connective tissue or rheumatic disease | 1.28 (0.83 – 1.97) | 0.26 |
| Peptic ulcer disease | 1.17 (0.78 – 1.74) | 0.45 |
| Mild liver disease | 1.26 (0.96 – 1.65) | 0.10 |
| Uncomplicated diabetes | 1.55 (1.24 – 1.93) | <.0001 |
| Diabetes with complications | 1.33 (1.05 – 1.69) | 0.0188 |
| Paraplegia or hemiplegia | 1.28 (0.87 – 1.89) | 0.21 |
| Renal disease | 1.42 (1.12 – 1.79) | 0.0040 |
| Cancer | 1.20 (0.89 – 1.63) | 0.23 |
| Moderate or severe liver disease | 2.90 (1.64 – 5.15) | 0.0003 |
| Metastatic cancer | 2.03 (1.26 – 3.27) | 0.0036 |
| AIDS | 1.47 (0.40 – 5.36) | 0.56 |
| Vaccinated | 0.53 (0.38 – 0.72) | <.0001 |
| Delta (ref: pre-delta) | 0.94 (0.68 – 1.30) | 0.71 |
| Omicron (ref: pre-omicron) | 0.65 (0.48 – 0.87) | 0.0035 |

**Table S2.** Independent predictors of inpatient outcomes for COVID-positive patients by the period of admission.

| Period | Pre-delta period | | Predominantly-Delta | | Predominantly-Omicron | |
| --- | --- | --- | --- | --- | --- | --- |
| **COVID pneumonia** | | | | | | |
| Predictor | OR (95% CI) | p | OR (95% CI) | p | OR (95% CI) | p |
| Age, per year | Na* | | 1.011 (1.000 - 1.021) | 0.0490 | 1.026 (1.019 - 1.033) | <.0001 |
| Male sex |  |  | 1.22 (0.87 - 1.72) | 0.26 | 1.51 (1.20 - 1.91) | 0.0005 |
| Morbid obesity |  |  | 2.36 (1.13 - 4.95) | 0.0230 | 1.61 (1.07 - 2.44) | 0.0236 |
| ECI, per 1 point |  |  | 0.999 (0.983 - 1.014) | 0.86 | 1.012 (1.002 - 1.023) | 0.0192 |
| Vaccinated |  |  | 0.29 (0.2 - 0.43) | <.0001 | 0.36 (0.28 - 0.46) | <.0001 |
| **Inpatient mortality** | | | | | | |
| Predictor | OR (95% CI) | p | OR (95% CI) | p | OR (95% CI) | p |
| Age, per year | 1.05 (1.042 - 1.058) | <.0001 | 1.005 (0.989 - 1.022) | 0.51 | 1.022 (1.008 - 1.037) | 0.0020 |
| Male sex | 1.65 (1.33 - 2.04) | <.0001 | 1.23 (0.73 - 2.07) | 0.45 | 1.35 (0.87 - 2.09) | 0.19 |
| Morbid obesity | 2.69 (1.84 - 3.94) | <.0001 | 0.54 (0.16 - 1.84) | 0.32 | 1.40 (0.61 - 3.23) | 0.43 |
| ECI, per 1 point | 1.065 (1.055 - 1.074) | <.0001 | 1.064 (1.041 - 1.087) | <.0001 | 1.054 (1.037 - 1.072) | <.0001 |
| Vaccinated | 0.34 (0.13 - 0.90) | 0.0297 | 0.36 (0.20 - 0.66) | 0.0010 | 0.9 (0.57 - 1.42) | 0.64 |
| **Length of stay (days)** | | | | | | |
| Predictor | Beta ± SE | p | Beta ± SE | p | Beta ± SE | p |
| Age, per year | 0.009 ± 0.010 | 0.36 | -0.026 ± 0.025 | 0.30 | 0.017 ± 0.010 | 0.08 |
| Male sex | 2.42 ± 0.31 | <.0001 | 0.75 ± 0.82 | 0.36 | 0.78 ± 0.34 | 0.0215 |
| Morbid obesity | 1.09 ± 0.56 | 0.05 | -1.14 ± 1.44 | 0.43 | 2.17 ± 0.60 | 0.0003 |
| ECI, per 1 point | 0.297 ± 0.016 | <.0001 | 0.307 ± 0.038 | <.0001 | 0.080 ± 0.015 | <.0001 |
| Vaccinated | -2.35 ± 1.33 | 0.08 | -1.52 ± 0.92 | 0.10 | -1.49 ± 0.35 | <.0001 |
| **Admission to ICU** | | | | | | |
| Predictor | OR (95% CI) | p | OR (95% CI) | p | OR (95% CI) | p |
| Age, per year | 1.002 (0.998 - 1.006) | 0.25 | 0.983 (0.972 - 0.994) | 0.0019 | 0.994 (0.985 - 1.003) | 0.16 |
| Male sex | 1.82 (1.60 - 2.08) | <.0001 | 1.41 (0.98 - 2.03) | 0.06 | 1.44 (1.05 - 1.96) | 0.0237 |
| Morbid obesity | 1.73 (1.38 - 2.18) | <.0001 | 0.94 (0.49 - 1.83) | 0.86 | 1.68 (1.02 - 2.77) | 0.0407 |
| ECI, per 1 point | 1.047 (1.041 - 1.054) | <.0001 | 1.059 (1.042 - 1.077) | <.0001 | 1.026 (1.013 - 1.039) | <.0001 |
| Vaccinated | 0.34 (0.18 - 0.64) | 0.0009 | 0.85 (0.57 - 1.27) | 0.42 | 0.88 (0.64 - 1.20) | 0.41 |
| **Invasive mechanical ventilation** | | | | | | |
| Predictor | OR (95% CI) | p | OR (95% CI) | p | OR (95% CI) | p |
| Age, per year | 0.994 (0.988 – 1.000) | 0.0386 | 0.977 (0.963 - 0.99) | 0.0006 | 0.992 (0.979 - 1.005) | 0.22 |
| Male sex | 1.99 (1.63 - 2.41) | <.0001 | 1.56 (1.00 - 2.43) | 0.05 | 1.48 (0.95 - 2.30) | 0.09 |
| Morbid obesity | 1.66 (1.21 - 2.29) | 0.0019 | 0.92 (0.41 - 2.08) | 0.84 | 1.69 (0.83 - 3.46) | 0.15 |
| ECI, per 1 point | 1.073 (1.064 - 1.082) | <.0001 | 1.074 (1.054 - 1.095) | <.0001 | 1.054 (1.037 - 1.072) | <.0001 |
| Vaccinated | 0.64 (0.29 - 1.41) | 0.27 | 0.55 (0.33 - 0.90) | 0.0185 | 0.75 (0.48 - 1.16) | 0.19 |

* The ICD-10 code for COVID pneumonia was introduced by CMS on 1/1/2021 and was not universally adopted during pre-Delta period. Abbreviations: ECI, Elixhauser’s comorbidity index.

**Table S3**. COVID-19 inpatients by the age group and the period of admission.

| Age group | Delta | Omicron | p | Delta and Omicron periods |
| --- | --- | --- | --- | --- |
| <55 years | 364 (42.3%) | 539 (34.6%) | 0.0042 | 903 (37.4%) |
| 55-64 years | 136 (15.8%) | 278 (17.9%) |  | 414 (17.1%) |
| 65-74 years | 154 (17.9%) | 290 (18.6%) |  | 444 (18.4%) |
| 75-84 years | 123 (14.3%) | 263 (16.9%) |  | 386 (16.0%) |
| ≥85 years | 83 (9.6%) | 186 (11.9%) |  | 269 (11.1%) |

**Figure S1.** Distribution of COVID-positive admissions by calendar month.


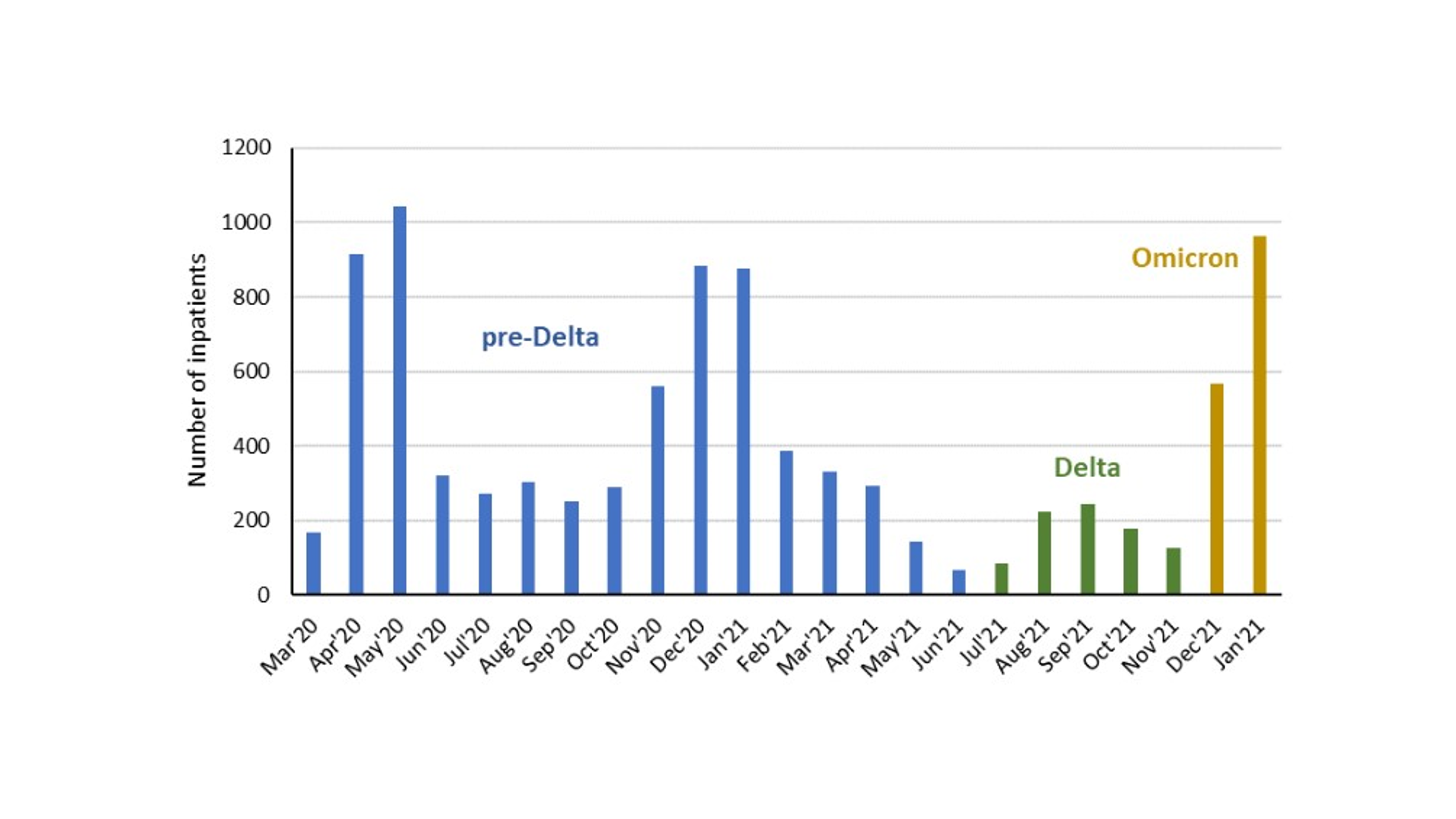

Supplement: Supplementary file 1 — Additional file 1: Table S1. Independent predictors of inpatient mortality among COVID-positive patients adjusted for individual comorbidities. Table S2. Independent predictors of inpatient outcomes for COVID-positive patients by the period of admission. Table S3. COVID-19 inpatients by the age group and the period of admission. Figure S1. Distribution of COVID-positive admissions by calendar month. [file 12879_2022_7657_MOESM1_ESM.docx]
